# Novel choledochojejunostomy technique “T‐shaped anastomosis” for preventing the development of postoperative cholangitis in pancreatoduodenectomy: A propensity score matching analysis

**DOI:** 10.1002/ags3.12744

**Published:** 2023-09-28

**Authors:** Nana Kimura, Takamichi Igarashi, Kenta Murotani, Ayaka Itoh, Toru Watanabe, Katsuhisa Hirano, Haruyoshi Tanaka, Kazuto Shibuya, Isaku Yoshioka, Tsutomu Fujii

**Affiliations:** ^1^ Department of Surgery and Science, Faculty of Medicine, Academic Assembly University of Toyama Toyama Japan; ^2^ Biostatistics Center, Graduate School of Medicine Kurume University Kurume Japan

**Keywords:** cholangitis, choledochojejunostomy, pancreatoduodenectomy, postoperative complication, T‐shaped anastomosis

## Abstract

**Background:**

There have been few studies of countermeasures against postoperative cholangitis, a serious complication after pancreaticoduodenectomy (PD) that impairs quality of life.

**Objective:**

To evaluate our recently developed, novel method of choledochojejunostomy with a larger anastomotic diameter, the “T‐shaped anastomosis.”

**Methods:**

The study included 261 cases of PD. The T‐shaped choledochojejunostomy technique was performed with an additional incision for a distance greater than half the diameter of the bile duct at the anterior wall of the bile duct and the anterior wall of the elevated jejunum. To compensate for potential confounding biases between the standard anastomosis group (*n* = 206) and the T‐shaped anastomosis group (*n* = 55), we performed propensity score matching (PSM). The primary endpoint was the incidence of medium‐term postoperative cholangitis adjusted for PSM.

**Results:**

In the PSM analysis, 54 patients in each group were matched, and the median bile duct diameter measured by preoperative CT was 8.8 mm versus 9.3 mm, the rate of preoperative biliary drainage was 31% versus 37%, the incidence of cholangitis within 1 month before surgery was 9% versus 13%, and the incidence of postoperative bile leakage was 2% versus 2%, with no significant differences. The incidence of medium‐term postoperative cholangitis was 15% versus 4%, and multivariate logistic regression revealed that T‐shaped choledochojejunostomy was an independent predictor of a reduced incidence of cholangitis (odds ratio, 0.17, 95% CI 0.02–0.81; *p* = 0.024).

**Conclusions:**

The T‐shaped choledochojejunostomy technique was shown to be effective with a significant reduction in the incidence of medium‐term postoperative cholangitis. Clinical trial identification: UMIN000050990.

## Graphical abstract

We developed a novel method of choledochojejunostomy with a larger anastomotic diameter, the “T‐shaped anastomosis,” in pancreatoduodenectomy. This technique was shown to be effective with a significant reduction in the incidence of medium‐term postoperative cholangitis.

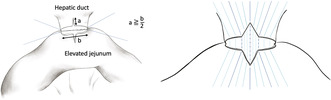

## 1 INTRODUCTION

Pancreatoduodenectomy (PD) is a demanding procedure that, among gastrointestinal surgeries, requires a high level of skill and appropriate perioperative management.^1^, ^2^, ^3^, ^4^, ^5^ Pancreatic cancer accounts for the majority of diseases requiring pancreatectomy. In recent years, advances in multidisciplinary treatment for pancreatic cancer have led to an increase in the 5‐year survival rate of pancreatic cancer patients and an increase in the number of patients who survive for a long period.^6^, ^7^, ^8^, ^9^ Therefore, we need to address mid‐ to long‐term complications after PD for stable and reliable continuation of postoperative chemotherapy. However, various complications, such as pancreatic fistula and delayed gastric emptying, can occur in 10%–23% of patients after PD^10^, ^11^ and overwhelmingly cause the most frequent and troublesome aspects of all abdominal surgeries.

One of the main complications occurring in the medium term after PD is cholangitis. Reportedly, cholangitis occurs in 9.5%–18.6% of PD patients.^12^, ^13^, ^14^ This is a complication that requires multiple hospitalizations and is sometimes fatal. Repeated postoperative cholangitis may impair the continuation of postoperative chemotherapy, possibly resulting in worsening of the prognosis of cancer. There have been several reports on risk factors for cholangitis after PD. Reported risk factors for cholangitis include preoperative small bile duct diameter, high BMI, male sex, preoperative biliary drainage, continuous suture of bile duct jejunal anastomosis, postoperative hepatolithiasis, and postoperative anastomotic stricture.^15^, ^16^, ^17^, ^18^ However, these factors are often not addressed by preoperative intervention. In this era of multidisciplinary treatment, we need specific techniques to decrease postoperative cholangitis; however, no novel anastomotic methods other than end‐to‐side choledochojejunostomy by interrupted suture have been reported.

We developed a novel method of choledochojejunostomy to enlarge the anastomotic diameter. In this study, we compared its usefulness for preventing postoperative cholangitis, as well as the incidence of postoperative complications, with those of conventional anastomosis methods using propensity score matching.

## 2 METHODS

### 2.1 Patients

A total of 272 patients underwent PD as an elective operation at Toyama University Hospital from April 2017 to June 2022. Among them, six patients were excluded because of hilar recurrence postoperatively that was diagnosed by computed tomography (CT) or magnetic resonance imaging (MRI) during postoperative follow‐up. In addition, we excluded five patients who had postoperative complications of Clavien–Dindo Grade IVb or higher because of possible effects on the medium‐ and long‐term postoperative course. Note that none of the complications in these five excluded patients were related to the choledochojejunostomy. Finally, 261 patients were included in this study, none of whom underwent R2 surgery.

The study was reviewed and approved (ref. No. R2021142) by the institutional review board and complied with the Strengthening the Reporting of Observational Studies in Epidemiology (STROBE) guidelines.^19^ All procedures in this study were performed in accordance with the guidelines of the Declaration of Helsinki. Written informed consent for treatment was obtained from each patient prior to the start of treatment, and consent for the use of data for research was obtained on an opt‐out basis.

### 2.2 Surgical procedure

We performed subtotal stomach‐preserving PD with a midline abdominal incision from the xiphoid to below the navel as a standard procedure. Concurrent superior mesenteric vein (SMV)/portal vein (PV) resection was performed if necessary.^20^, ^21^ The bile duct was temporarily clamped with a bulldog vascular clamp to prevent potential intraabdominal infection from the bile juice. The bile duct was subsequently cut with scissors, and its stump was measured. Bile juice was collected for intraoperative culture. We performed the modified Child reconstruction. The jejunal limb was brought up to the pancreatic and bile duct stumps via a retrocolic route. Pancreatojejunostomy was performed by the modified Blumgart method in all patients.^22^ In the soft pancreas, the stent tube was inserted into the main pancreatic duct, and the other side of the tube exited the body. Gastrojejunostomy was performed by hand stitching using a 3–0 synthetic absorbable blade filament and 4–0 synthetic absorbable monofilament or a mechanical anastomosis with a surgical stapling device, and Braun anastomosis was added in all patients.

The drainage tubes were placed on the ventral and dorsal sides of the pancreatojejunostomy and the dorsal side of the choledochojejunostomy. All patients received prophylactic antibiotics for 3 days and a proton pump inhibitor to prevent stress peptic ulcers.^23^ We usually used cefazoline as the prophylactic antibiotic; however, the selection of antibiotics was changed according to preoperative bile culture if the endoscopic nasobiliary drainage tube was placed preoperatively.

### 2.3 Choledochojejunostomy

Choledochojejunostomy was performed with an interrupted suture using 5–0 synthetic absorbable monofilament sutures in an end‐to‐side fashion. Intraoperative biliary drainage tubes were not routinely used, and biliary drainage tubes were inserted only if there was postoperative concern of biliary leakage during choledochojejunostomy.

**FIGURE 1:**
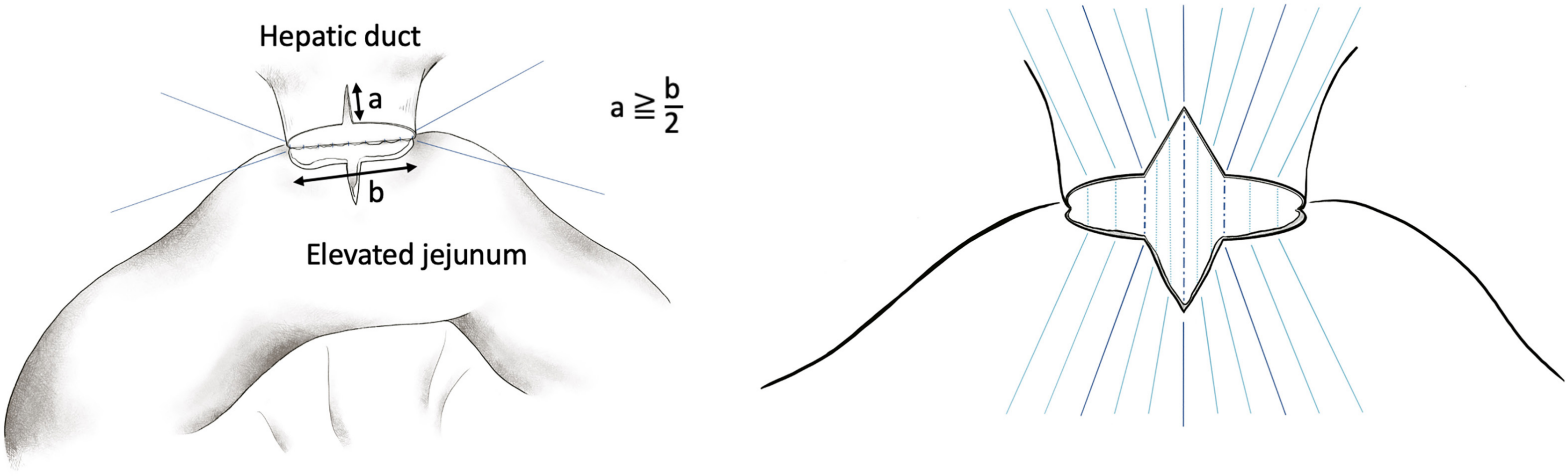
Scheme of T‐shaped anastomosis.

T‐shaped anastomosis was exclusively performed starting in March 2021. The method of T‐shaped anastomosis was as follows (Figure 1). First, the diameter of the resection stump of the bile duct is measured. The dorsal wall is sutured with a 5–0 synthetic absorbable monofilament interrupted suture as in a conventional choledochojejunostomy. Once the dorsal wall is sutured, a slit of length greater than half the diameter of the bile duct is added to the anterior median surface of the hepatic duct and the elevated jejunum. An interrupted suture is made, including the apex of the incision and the two points on either side of the incision. The number of interrupted sutures depends on the diameter of the bile duct, but usually 11 sutures are needed for the anterior wall.

Except for patients for whom the bile duct was cut to the very edge of the porta hepatis, preventing T‐shaped anastomosis, and those for whom the bile duct was formed with two holes, T‐shaped anastomosis has been performed for all patients since the inception of the technique whenever technically possible, even in cases of preoperative cholangitis or biliary drainage.

### 2.4 Postoperative complications

All preoperative complications were graded according to the Clavien–Dindo classification.^24^ Postoperative cholangitis was diagnosed when the criteria for suspicion or definition were met according to the Tokyo Guidelines 2018, specifically fever, laboratory data, jaundice, biliary dilatation, and imaging such as biliary stricture.^25^ Liver abscesses and biliary stones were diagnosed by either CT or MRI. Benign biliary stenosis was defined as suspected stenosis on CT or MRCP and endoscopically confirmed stenosis. The severity of cholangitis was also diagnosed according to the severity criteria of the Tokyo Guidelines 2018.^25^ In this study, we categorized cholangitis as short‐ (within 30 days postoperatively) and medium‐term (31 days to 18 months postoperatively).

### 2.5 Evaluated factors

The primary endpoint of this study was the incidence of medium‐term postoperative cholangitis adjusted for propensity score. Clinical data were collected retrospectively for all patients and included patient demographics (age, sex, height, weight, body mass index [BMI]), preoperative and postoperative diagnosis, pathological examination, perioperative clinical information, and complications. Body temperature, white blood cell counts, C‐reactive protein (CRP), aspartate aminotransferase (AST), alanine aminotransferase (ALT), alkaline phosphatase (ALP), γ‐glutamyl transpeptidase (γ‐GT), and total bilirubin (T‐Bil) were measured before and after surgery and when cholangitis was suspected. We measured the common hepatic duct diameter by using a CT imaging scale before surgery. The postoperative observational period was terminated at 18 months (540 days) since medium‐term postoperative cholangitis as the primary endpoint was defined as up to 18 months. With regard to postoperative follow‐up, postoperative patients with malignant tumors were seen at the hospital once a month, and patients with low‐grade tumors were seen every 3 months. When patients were being followed at other hospitals, the physicians at those hospitals were asked to carefully check for the presence of cholangitis.

### 2.6 Statistical analysis

A biostatistician (K.M.) was responsible for the statistical analysis. Differences in the nominal data between the two groups were examined using the chi‐squared test or Fisher's exact test when the expected value was <5. Differences in quantitative variables were evaluated using Student's *t* test or the Mann–Whitney *U* test if the distribution was abnormal. All cutoff values for the development of medium‐term postoperative cholangitis were determined using the Youden index.

To reduce the effect of selection bias, propensity score analysis was performed using the following covariates: age, BMI, previous abdominal surgery, common hepatic duct diameter on preoperative CT, primary disease (pancreatic cancer/other), preoperative biliary drainage, and preoperative cholangitis. These seven variables have been identified as clinicopathologic factors that could contribute to the development of cholangitis.^15^, ^16^, ^17^, ^18^ The Kaplan–Meier method was used to compare the accumulated incidence of medium‐term postoperative cholangitis between the standard anastomosis group and the T‐shaped anastomosis group. We used univariate and multivariate logistic regression to generate odds ratios (ORs), including 95% CIs, for clinical factors that would predict medium‐term postoperative cholangitis. Variables included in the multivariate models had a *p* value of 0.1 or less in the univariate analysis. In addition to 1:1 matching of the standard anastomosis group and the T‐shaped anastomosis group, a 2:1‐matching, inverse‐probability‐of‐treatment‐weighting analytical method was performed as a sensitivity analysis.

A *p* value <0.05 was considered to indicate statistical significance. All statistical analyses were performed using JMP statistical software (version 15.0; SAS Institute).

## 3 RESULTS

### 3.1 Characteristics and preoperative status of all unmatched patients

The characteristics, preoperative status, and preoperative blood test results of all patients (*n* = 261) are summarized in Table 1. In the comparison of the standard anastomosis group (*n* = 206) versus the T‐shaped anastomosis group (*n* = 55) before propensity score matching (PSM), the median age was 72 versus 73 years, the male/female ratio was 127/79 versus 33/22, the median BMI was 22.2 versus 21.7, the median common hepatic duct diameter measured by preoperative CT was 8.9 mm versus 9.2 mm, the rate of preoperative biliary drainage was 37% versus 36%, and the incidence of cholangitis within 1 month before surgery was 10% versus 15%, with no significant differences. The two groups did not differ significantly in terms of preoperative comorbidities, including a history of diabetes mellitus. The preoperative albumin level and total lymphocyte count were also similar in both groups.

**TABLE 1:** Comparison of clinical characteristics between the standard anastomosis group and the T‐shaped anastomosis groups before propensity score matching.

Variable	Standard anastomosis	T‐shaped anastomosis	*p*
	*n* = 206	*n* = 55	
Background			
Age (year)^a^	72 (27–90)	73 (15–84)	0.843
Sex (male/female)	127/79	33/22	0.823
Body mass index (kg/m^2^)^a^	22.2 (15.2–41)	21.7 (15.5–27.9)	0.152
Primary disease (pancreatic cancer/others)	68/138	33/22	<0.001
Preoperative status			
Common hepatic duct diameter on preoperative CT (mm)^a^	8.9 (3.5–33.6)	9.2 (4.4–22.8)	0.659
Preoperative biliary drainage (yes/no)	77/129	20/35	0.890
Preoperative cholangitis (yes/no)	21/185	8/47	0.362
Previous abdominal surgery (yes/no)	73/133	21/34	0.706
Neoadjuvant treatment (yes/no)	41/165	33/22	<0.001
Preoperative PNI^a^	45.4 (26.3–58.5)	43.6 (27.4–55.3)	0.093
Operative factor			
Pancreatic texture (soft/hard)	144/62	27/28	0.004
Laparoscopic surgery	1	1	0.313
Combined vascular resection	17	16	<0.001
Operative time (min)^a^	509.5 (230–945)	572 (220–804)	0.033
Blood loss volume (mL)^a^	502.5 (60–2750)	740 (50–2345)	0.014
Intraoperative blood transfusion (yes/no)	30/176	14/41	0.066
Intraoperative biliary drainage tube placement (yes/no)	17/189	2/53	0.242
Postoperative factor			
AST peak value (U/L)^a^	92 (13–1435)	92 (12–612)	0.726
ALT peak value (U/L)^a^	87 (8–1775)	79 (19–670)	0.140
ALP peak value (U/L)^a^	136 (9–975)	144 (80–440)	0.210
γGT peak value (U/L)^a^	139 (16–983)	135 (26–668)	0.713
T‐Bil peak value (U/L)^a^	1 (0.4–6.7)	0.9 (0.4–4.7)	0.833
Postoperative bile leakage (yes/no)	8/198	1/54	0.456
Clinically relevant pancreatic fistula (yes/no)	52/154	10/45	0.274
Delayed gastric emptying (yes/no)	11/195	6/49	0.137
Postoperative complications (yes/no)	71/135	16/39	0.453
Clavien–Dindo (≤II/≥IIIa)	126/80	39/16	0.183
Length of hospital stay (days)^a^	25 (9–112)	19 (11–86)	0.096
Postoperative short‐term cholangitis (within POD30) (yes/no)	4/202	1/54	0.953
Postoperative medium‐term cholangitis (POD31‐18 months) (yes/no)	27/179	2/53	0.047
Severity of cholangitis (grade I/II/III)^b^	15/7/5	0/2/0	0.092
Repeated postoperative medium‐term cholangitis (POD31‐18 months) (yes/no)	13/193	1/54	0.189
Postoperative benign biliary stenosis (yes/no)	9/197	0/55	0.033
Need for endoscopic intervention (yes/no)	13/193	0/55	0.056
Postoperative observational period^a^	531.5 (25–540)	377 (22–540)	0.008

Abbreviations: ALP, alkaline phosphatase; ALT, alanine aminotransferase; AST, aspartate aminotransferase; PNI, prognostic nutritional index; POD, postoperative day; T‐Bil, total bilirubin; γGT, γ‐glutamyl transpeptidase.

^a^ Median (range).

^b^ According to Tokyo Guidelines 18.

There was a significant difference in the ratio of pancreatic cancer/other as the primary disease: 68/138 versus 33/22 (*p* < 0.001); therefore, there was a significant difference between the two groups in the percentage of patients who received preoperative treatment (41/165 vs. 33/22, *p* < 0.001).

### 3.2 Perioperative details and postoperative complications in unmatched patients

The proportion of patients who underwent laparoscopic surgery was 0.5% in the standard anastomosis group and 1.8% in the T‐shaped anastomosis group, with no significant differences. Because pancreatic cancer was more common in the T group of unmatched patients, the median main pancreatic duct diameter (3 [1–20] vs. 4 [1–25] mm, respectively; *p* = 0.007) and median operative time (509.5 [230–945] vs. 572 [220–804] min, respectively; *p* = 0.033) were greater in the T‐shaped anastomosis group. However, the frequency of intraoperative blood transfusions was not significantly different between the two groups.

Intraoperative retrograde transhepatic biliary drainage tube placement was 8.3% versus 3.8%, with no significant differences. Postoperative peak values of hepatobiliary enzymes such as AST, ALT, ALP, γ‐GT, and T‐Bil after surgery were not significant in either group. With regard to postoperative complications, the incidence of bile leakage was 3.9% versus 1.8%, that of clinically relevant pancreatic fistula was 25% versus 18%, that of delayed gastric emptying was 5.3% versus 10.9%, and the median length of hospital stay was 25 versus 19 days, with no significant differences. The median postoperative observational period significantly differed between the two groups (531.5 vs. 377 days, respectively; *p* = 0.008) since the T‐shaped anastomosis was performed more recently. The incidence of short‐term postoperative cholangitis was 1.9% in the standard anastomosis group and 1.8% in the T‐shaped anastomosis group, and the incidence values of medium‐term postoperative cholangitis in the standard and T‐shaped anastomosis groups were 13% and 3.6%, respectively. The average number of times cholangitis occurred was 2 (1–6) versus 1.5 (1, 2), respectively. The severity of cholangitis was mild in 15 cases, moderate in seven cases, and severe in five cases in the standard anastomosis group and moderate in two cases in the T‐shaped anastomosis group. All 15 patients with mild cholangitis in this study had a spike fever of 38°C or higher, and all required hospitalization for treatment. In addition, some patients with malignant tumors had to discontinue adjuvant chemotherapy because of cholangitis.

Although cases of hilar recurrence were excluded from the present study, no cases of cholangitis developed in any patients with hilar recurrence. In addition, there were no cases of postoperative cholangitis in patients with early recurrence in the liver.

Postoperative benign biliary stricture occurred in nine of 27 (33%) patients in the standard anastomosis group and zero of two patients in the T‐shaped anastomosis group. Endoscopic interventions were performed in 13 of 27 (48%) patients with cholangitis in the standard anastomosis group. Of the 13 patients who underwent endoscopic intervention, only three cases did not require endoscopic intervention because there was no anastomotic stricture or intrahepatic calculus. In the other 10 cases, all patients underwent endoscopic intervention, of which five patients underwent endoscopic biliary drainage and eight patients underwent endoscopic balloon dilation. Intrahepatic stones were treated with lithotripsy in four cases, three of which required more than two lithotripsy procedures. In endoscopic intervention, there was overlap in several cases.

The average number of endoscopic interventions was 2.7, with a maximum of seven interventions needed. Two patients with cholangitis in the T‐shaped anastomosis group did not undergo endoscopy.

### 3.3 Analysis of propensity score‐matched patients

**FIGURE 2:**
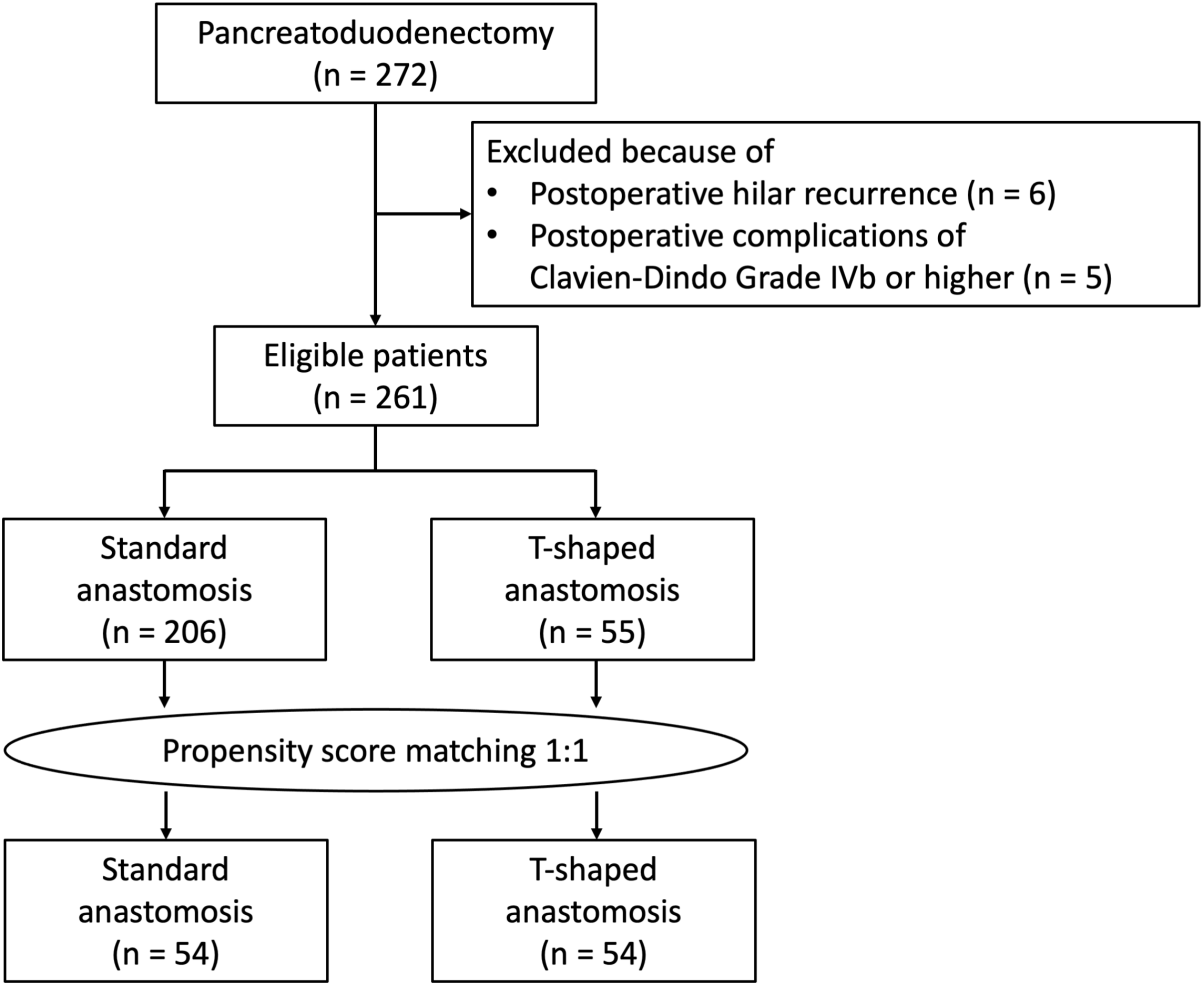
Flow diagram of patient enrollment.

**FIGURE 3:**
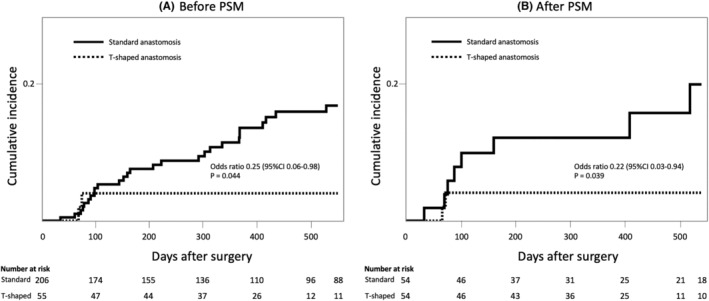
Cumulative incidence of medium‐term postoperative cholangitis (A) before PSM and (B) after PSM. CI, confidence interval; PSM, propensity score matching.

To reduce the impact of selection bias, PSM was performed using seven selected baseline characteristics, and 54 patients in each group were matched (Figure 2). In Table 2, the primary disease ratio (pancreatic cancer/other) was 34/20 versus 32/22, the common hepatic duct diameter measured by preoperative CT was 8.8 mm versus 9.3 mm, the rate of preoperative biliary drainage was 31% versus 37%, and the incidence of cholangitis within 1 month before surgery was 9% versus 13%, with no significant differences between the standard anastomosis group and the T‐shaped anastomosis group. Significant differences in operative time and blood loss disappeared after PSM. The frequency of intraoperative biliary drainage tube placement was similar. The incidence of postoperative bile leakage was 1.9% versus 1.9%, the incidence of postoperative complications (Clavien–Dindo grade IIIa or higher) was 33% versus 30%, and the median length of hospital stay was 24.5 versus 19 days, with no significant differences. The postoperative observational period was also not significantly different between the two groups (395.5 vs. 370.5 days, respectively; *p* = 0.476); however, the incidence of medium‐term postoperative cholangitis was significantly lower in the T‐shaped anastomosis group than in the standard anastomosis group (3.7% vs. 14.8%, respectively; *p* = 0.046). The cumulative incidence analysis similarly showed significantly lower cholangitis in the T‐shaped anastomosis group (Figure 3). The mean number of times that cholangitis recurred was 1.6 (1–3) among the eight patients in the standard anastomosis group and 1.5 (1, 2) among the two patients in the T‐shaped anastomosis group. Endoscopy intervention was performed in four of the eight cholangitis patients in the standard anastomosis group, all of whom also underwent balloon dilatation and other interventions. The mean number of interventions was two (1–4). No endoscopy was performed in two patients in the T‐shaped anastomosis group. After PSM, benign biliary stenosis and endoscopic intervention were significantly less common in the T‐shaped anastomosis group.

**TABLE 2:** Comparison of clinical characteristics between the standard anastomosis group and the T‐shaped anastomosis groups after propensity score matching.

Variable	Standard anastomosis	T‐shaped anastomosis	*p*
	*n* = 54	*n* = 54	
Background			
Age (year)^a^	71 (44–85)	73 (15–84)	0.631
Sex (male/female)	28/26	33/21	0.332
Body mass index (kg/m^2^)^a^	22.2 (16.8–32.3)	21.7 (15.5–27.9)	0.403
Primary disease (pancreatic cancer/others)	34/20	32/22	0.693
Preoperative status			
Common hepatic duct diameter on preoperative CT (mm)^a^	8.8 (3.8–20.8)	9.3 (4.4–22.8)	0.380
Preoperative biliary drainage (yes/no)	17/37	20/34	0.543
Preoperative cholangitis (yes/no)	5/49	7/47	0.540
Previous abdominal surgery (yes/no)	20/34	20/34	1
Neoadjuvant treatment (yes/no)	23/31	32/22	0.083
Preoperative PNI	6/48	6/48	1
Operative factor			
Pancreatic texture (soft/hard)	29/25	26/28	0.564
Laparoscopic surgery	0	1	0.315
Combined vascular resection	9	16	0.108
Operative time (min)^a^	496 (249–911)	572 (220–804)	0.258
Blood loss volume (mL)^a^	565 (110–2130)	740 (50–2345)	0.125
Intraoperative blood transfusion (yes/no)	6/48	13/41	0.074
Intraoperative biliary drainage tube placement (yes/no)	2/52	2/52	1
Postoperative factor			
AST peak value (U/L)^a^	90 (22–1379)	93 (12–612)	0.296
ALT peak value (U/L)^a^	70 (21–1686)	80 (19–670)	0.849
ALP peak value (U/L)^a^	141 (64–426)	147 (80–440)	0.647
γGT peak value (U/L)^a^	138 (16–612)	136 (26–668)	0.922
T‐Bil peak value (U/L)^a^	0.9 (0.4–4.5)	0.9 (0.4–4.7)	0.998
Postoperative bile leakage (yes/no)	1/53	1/53	1
Clinically relevant pancreatic fistula (yes/no)	15/39	10/44	0.254
Delayed gastric emptying (yes/no)	2/52	6/48	0.142
Postoperative complications (yes/no)	18/36	15/39	0.531
Clavien–Dindo (≤II/≥IIIa)	36/18	38/16	0.679
Length of hospital stay (days)^a^	24.5 (11–85)	19 (11–86)	0.368
Postoperative short‐term cholangitis (within POD30) (yes/no)	0/54	1/53	0.315
Postoperative medium‐term cholangitis (POD31‐18 months) (yes/no)	8/46	2/52	0.046
Severity of cholangitis (grade I/II/III)^b^	6/1/1	0/2/0	0.054
Repeated postoperative medium‐term cholangitis (POD31‐18 months) (yes/no)	3/51	1/53	0.308
Postoperative benign biliary stenosis (yes/no)	5/49	0/54	0.022
Need for endoscopic intervention (yes/no)	4/50	0/54	0.042
Postoperative observational period^a^	395.5 (33–540)	370.5 (22–540)	0.513

Abbreviations: ALP, alkaline phosphatase; ALT, alanine aminotransferase; AST, aspartate aminotransferase; PNI, prognostic nutritional index; POD, postoperative day; T‐Bil, total bilirubin; γGT, γ‐glutamyl transpeptidase.

^a^ Median (range).

^b^ According to Tokyo Guidelines 18.

### 3.4 Factors predicting medium‐term postoperative cholangitis

The factors predicting medium‐term postoperative cholangitis in propensity score‐matched patients are shown in Table 3. Univariate logistic regression with propensity scores showed that higher postoperative peak values of AST and ALT and longer postoperative hospital stay were significantly associated with the onset of medium‐term postoperative cholangitis and that medium‐term postoperative cholangitis was significantly less frequent in the T‐shaped anastomosis group than in the standard anastomosis group. Multivariate analysis showed that T‐shaped choledochojejunostomy was an independent predictor of a reduced incidence of cholangitis (odds ratio, 0.17; 95% confidence interval, 0.02–0.81; *p* = 0.024) (Table 4).

**TABLE 3:** Univariate logistic regression of the predictive factors for postoperative medium‐term cholangitis after propensity score matching.

	*n*	OR	95% CI	*p*
Background				
Age (years)		0.99	0.93–1.06	0.684
Sex				
Male	61	1.17	0.32–4.83	0.813
Female	47			
Body mass index (kg/m^2^)		0.85	0.65–1.07	0.168
Primary disease				
Pancreatic cancer	66	1.54	0.40–7.49	0.539
Others	42			
Preoperative status				
Common hepatic duct diameter on preoperative CT				
<6.4 mm	25	1.48	0.30–5.82	0.600
≥6.4 mm	83			
Preoperative biliary drainage				
Yes	37	1.31	0.32–4.92	0.699
No	71			
Preoperative cholangitis				
Yes	12	0.88	0.05–5.38	0.905
No	96			
Neoadjuvant treatment				
Yes	55	0.61	0.15–2.28	0.467
No	53			
Preoperative PNI				
<43.15	47	0.53	0.11–2.01	0.357
≥43.15	61			
Operative factor				
Pancreatic texture				
Soft	55	0.96	0.25–3.65	0.951
Hard	53			
T‐shaped anastomosis				
Yes	54	0.22	0.03–0.94	0.039
No	54			
Combined vascular resection				
Yes	25	0.82	0.16–4.11	0.805
No	83			
Operative time				
≥545 min	59	0.52	0.14–1.96	0.336
<545 min	49			
Blood loss volume				
≥705 mL	53	0.41	0.10–1.68	0.217
<705 mL	55			
Intraoperative blood transfusion				
Yes	19	0.49	0.06–4.15	0.516
No	89			
Intraoperative biliary drainage tube placement				
Yes	4	3.13	0.15–27.26	0.386
No	104			
Postoperative factor				
AST peak value				
≥78 U/L	64	3.89	0.04–1.04	0.058
<78 U/L	44			
ALT peak value				
≥100 U/L	39	4.19	0.06–0.82	0.022
<100 U/L	69			
ALP peak value				
≥141 U/L	58	0.58	0.51–6.19	0.376
<141 U/L	50			
γ‐GT peak value				
≥160 U/L	44	0.45	0.09–1.61	0.226
<160 U/L	64			
T‐Bil peak value				
≥1.4 mg/dL	30	0.44	0.67–8.96	0.193
<1.4 mg/dL	78			
Postoperative complications				
Yes	75	1.85	0.08–2.31	0.430
No	33			
Postoperative bile leakage				
Yes	2	8.64	0.33–228.91	0.166
No	106			
Clinically relevant pancreatic fistula				
Yes	25	1.12	0.23–4.14	0.873
No	83			
Delayed gastric emptying				
Yes	8	1.16	0.06–7.42	0.898
No	100			
Clavien–Dindo				
≥IIIa	34	0.93	0.19–3.58	0.915
≤II	74			
Length of hospital stay				
≥37 days	25	3.90	0.99–15.33	0.070
<37 days	83			
Postoperative observational period				
≥344 days	63	3.13	0.63–15.49	0.163
<344 days	45			

Abbreviations: ALP, alkaline phosphatase; ALT, alanine aminotransferase; AST, aspartate aminotransferase; CI, confidence interval; OR, odds ratio; PNI, prognostic nutritional index; T‐Bil, total bilirubin; γ‐GT, γ‐glutamyl transpeptidase.

**TABLE 4:** Multivariate analysis for postoperative medium‐term cholangitis in patients who underwent PD.

Variable	Postoperative medium‐term cholangitis	
	Odds ratio (95% CI)	*p*
T‐shaped anastomosis	0.17 (0.02–0.81)	0.024
Postoperative peak AST (≥78)	0.58 (0.02–6.84)	0.668
Postoperative peak ALT (≥100)	5.55 (0.82–112.04)	0.083
Length of hospital stay (≥37 days)	2.78 (0.64–12.34)	0.170

Abbreviations: ALT, alanine aminotransferase; AST, aspartate aminotransferase; CI, confidence interval.

### 3.5 Sensitivity analysis

In Table 5, logistic regression between the standard anastomosis group (*n* = 106) and the T‐shaped anastomosis group (*n* = 53) using 2:1 PSM also showed that the incidence of medium‐term postoperative cholangitis was significantly lower in the T‐shaped anastomosis group (odds ratio, 0.26, 95% confidence interval 0.04–0.97; *p* = 0.045). In logistic regression of the inverse probability of treatment weighting, T‐shaped anastomosis significantly reduced the frequency of medium‐term cholangitis (odds ratio, 0.16, 95% confidence interval 0.06–0.35; *p* < 0.001).

**TABLE 5:** Odds ratios of postoperative medium‐term cholangitis in the T‐shaped anastomosis group compared with the standard anastomosis group.

	*n*	Event	Odds ratio	95% CI	*p*
Unadjusted	261	29 (11.1%)	0.25	0.04–0.87	0.027
IPTW	261	29 (11.1%)	0.16	0.06–0.35	<0.001
Matching (1:1)	108	10 (9.3%)	0.22	0.03–0.94	0.039
Matching (1:2)	159	16 (10.1%)	0.26	0.04–0.97	0.045

*Note*: Propensity score analysis was performed using the following covariates: age, body mass index, previous abdominal surgery, common hepatic duct diameter on preoperative CT, primary disease (pancreatic cancer/other), preoperative biliary drainage, and preoperative cholangitis.

Abbreviations: CI, confidence interval; IPTW, inverse probability of treatment weighting.

## 4 DISCUSSION

Postoperative cholangitis after PD is an extremely important and sometimes fatal complication.^12^, ^26^ Based on the TG13 guidelines, Okabayashi et al. reported cholangitis after choledochojejunostomy in 45 of 583 patients (7.7%), with anastomotic stricture in approximately 60% of the 45 patients.^27^ They showed that nonoperative management for stenosis, such as percutaneous balloon dilation or endoscopic stent insertion, provided improvement in more than 80% of patients, but surgical intervention was required in 15.4% of patients.^27^ Endoscopic intervention at the anastomotic site of choledochojejunostomy after PD remains an invasive procedure for the patient, although in recent years, the intervention has become more universal than before. Since a certain number of patients with postoperative cholangitis require surgery or develop severe organ failure (Clavien–Dindo classification ≥ Grade IIIa),^28^ the establishment of cholangitis prevention methods is an urgent issue. In many reports, researchers discuss predictive factors of the development of cholangitis after PD; however, its prevention is the focus of only a few reports.

To prevent postoperative cholangitis, it is important to ensure the passage of the bile duct jejunal anastomosis and elevated jejunum and to prevent the formation of sludge or stones due to bile stenosis. To avoid anastomotic stenosis, it is important to anastomose healthy bile duct tissue with adequate blood flow and without tension. Another option is to enlarge the anastomotic opening. Hiyoshi et al. reported the development of “hepaticoplasty,” an incision in the left wall of the common hepatic duct, as a cholangioplasty to prevent postoperative cholangitis.^28^ They made a 5–10‐mm incision in the left side of the common hepatic duct wall with an electrical scalpel, taking care not to injure the hepatic artery or the peribiliary vascular plexus. The reason for placing the incision on the left side of the hepatic duct is the anatomical factor that hepatolithiasis is more common in the left lobe.^29^ The left hepatic duct and the common hepatic duct meet at a sharp angle, which causes bile stasis, and by incising the bile duct wall in the area and widening the diameter of the bile duct, the biliary flow through the anastomotic portion to the intestine is smooth, preventing the occurrence of postoperative cholangitis.^30^

In this study, we developed a novel anastomotic technique called “T‐shaped anastomosis” for the prevention of postoperative cholangitis, which allows all surgeons to easily and reliably ensure a wide anastomotic diameter. T‐shaped anastomosis was performed after March 2021, and PD for pancreatic cancer tended to be more common during this period because there were fewer pancreatectomies for low‐grade malignant disease due to the COVID‐19 pandemic. To objectively evaluate the efficacy of T‐shaped anastomosis as an anastomotic technique, we used PSM to equalize the preoperative factors of the control and comparison groups as much as possible. The criteria for cholangitis also complied with the latest TG18 guidelines. Since there are no references in previous reports regarding when to evaluate cholangitis in the postoperative period, the timing was defined as within 18 months postoperatively in this study. The incidence of medium‐term postoperative cholangitis in the T‐shaped anastomosis group was significantly lower (4%) and was suggested to be an independent factor in the prevention of postoperative cholangitis. Although the number of stitches required was increased compared to the standard anastomosis, the operative time after PSM was comparable, and the associated complications, such as bile leakage, were not significantly different. This is the first study to demonstrate the usefulness of novel choledochojejunostomy by PSM.

Although the results of this study are interesting, our analysis had several limitations. First, this was a retrospective study based on single‐center data, leaving open the possibility that confounding factors and selection bias were included. Second, the limited sample size may have influenced our results. Third, as noted in the Methods section, T‐shaped anastomosis has been performed in all patients since March 2021, except for patients for whom the bile duct was cut up to the hilar region, which prevented creating the T‐shaped anastomosis, and those for whom the bile duct was formed with two holes. It cannot be denied that this may have resulted in a selection bias and reduced the frequency of cholangitis for T‐shaped anastomosis. Fourth, long‐term results, e.g., the incidence of cholangitis over a 5‐year period, need to be examined. Therefore, more studies with a higher level of evidence, such as multicenter randomized trials, are needed in the future.

## 5 CONCLUSIONS

In conclusion, the T‐shaped choledochojejunostomy technique was shown to be effective, with postoperative complications comparable to those of standard anastomosis and a significant reduction in the incidence of medium‐term postoperative cholangitis.

## AUTHOR CONTRIBUTIONS

Study concepts: Kimura, Igarashi, Fujii. Study design: Kimura, Igarashi, Shibuya, Yoshioka, Fujii. Data acquisition: Kimura, Itoh, Hirano. Quality control of data and algorithms: Tanaka, Hirano, Fujii. Data analysis and interpretation: Kimura, Igarashi, Murotani, Fujii. Statistical analysis: Kimura, Murotani, Fujii. Manuscript preparation: Kimura, Igarashi. Manuscript editing: Tanaka, Shibuya, Yoshioka. Manuscript review: Shibuya, Yoshioka, Fujii. All authors approved the final version of this manuscript.

## FUNDING INFORMATION

The authors have no sources of funding to declare.

## CONFLICT OF INTEREST STATEMENT

The authors declare no conflicts of interest for this article.

## ETHICS STATEMENT

Approval of the research protocol: The study was reviewed and approved (ref. No. R2021142) by the institutional review board and complied with the Strengthening the Reporting of Observational Studies in Epidemiology (STROBE) guidelines.^19^ All procedures in this study were performed in accordance with the guidelines of the Declaration of Helsinki.

Informed Consent: Written informed consent for treatment was obtained from each patient prior to the start of treatment, and consent for the use of data for research was obtained on an opt‐out basis.

Registry and the Registration No. of the study/trial: N/A.

Animal Studies: N/A.

## Data Availability

Data supporting the findings of this study are available on request from the corresponding author.
